# How the National Health Insurance Coverage policy changed the use of lenvatinib for adult patients with advanced hepatocellular carcinoma: a retrospective cohort analysis with real world big data

**DOI:** 10.1186/s12939-023-02052-9

**Published:** 2023-12-11

**Authors:** Yanyan Liu, Yuwen Bao, Yaxin Huang, Mengdie Zhang, Xin Li

**Affiliations:** 1 Nanjing Drum Tower Hospital Clinical College of Nanjing Medical University, Nanjing, China; 2 School of Health Policy and Management, Nanjing Medical University, Nanjing, China; 3 Department of Regulatory Science and Pharmacoeconomics, School of Pharmacy, Nanjing Medical University, Nanjing, China; 4 Center for Global Health, School of Public Health, Nanjing Medical University, Nanjing, China; 5 Nanjing Medical University, No.101 Longmian Avenue, Jiangning District, Nanjing, Jiangsu P.R. China

**Keywords:** The utilization of lenvatinib, Advanced hepatocellular carcinoma, The national health insurance coverage

## Abstract

**Background:**

To establish a long-term mechanism to control the cost burden of drugs, the Chinese government organized seven rounds of price negotiations for the national reimbursement drug list (NRDL) from 2016 to the end of 2022. The study aimed to evaluate the impact of the National Health Insurance Coverage (NHIC) policy on the use of lenvatinib as the first-line treatment for advanced hepatocellular carcinoma (HCC) within a specific medical insurance region from the micro perspective of individual patient characteristics.

**Methods:**

The data of HCC patients that received lenvatinib from September 2019 to August 2022 was retrieved from the Medical and Health Big Data Center and longitudinally analyzed. Contingency table chi-square statistics and binary logistic regression analysis were used to compare the differences in the categorical variables. Interrupted time-series (ITS) regression analysis was performed to evaluate the changes in the utilization of lenvatinib over 36 months. Multiple linear regression was used to analyze the impact of receiving lenvatinib on the total hospitalization expenses of hospitalized patients with advanced HCC.

**Results:**

A total of 12,659 patients with advanced HCC were included in this study. The usage rate of lenvatinib increased from 6.19% to 15.28% over 36 months (*P* < 0.001). By controlling the other factors, consistent with this, the probability of patients with advanced HCC receiving lenvatinib increased by 2.72-fold after the implementation of the NHIC policy (OR = 2.720, 95% CI:2.396–3.088, *P* < 0.001). Older, residency in rural areas, lack of fixed income, treatment at hospitals below the tertiary level, and coverage by urban-rural residents’ basic medical insurance (URRBMI) were the factors affecting the use of lenvatinib among patients with advanced HCC (*P* < 0.05). After the implementation of the NHIC policy, the total hospitalization expenses increased (Beta=-0.040, *P* < 0.001). However, compared to patients who received lenvatinib, the total hospitalization expenses were higher for those who did not receive the drug (US$5022.07 ± US$5488.70 vs. US$3701.63 ± US$4330.70, Beta = 0.062, *P* < 0.001).

**Conclusions:**

The NHIC policy has significantly increased the utilization of lenvatinib. In addition, we speculate that establishing multi-level medical insurance systems for economically disadvantaged patients would be beneficial in improving the effectiveness of the NHIC policy in the real world.

## Introduction

Globally, liver cancer is most prevalent in Southeast Asia, the Western Pacific region, and central African countries [1]. In China, primary liver cancer is the fourth most common cancer overall, and the second leading cause of cancer-related deaths. Over 410,000 new cases of liver cancer were diagnosed in China in 2020, accounting for 45.3% of cases worldwide, along with 391,000 related deaths that accounted for 47.1% of deaths worldwide [2]. Due to its high malignancy, insidious onset, and late diagnosis, the prognosis for liver cancer is poor and the 5-year survival rate is only 14.1% globally [3]. In addition, liver cancer also imposes a heavy personal and national economic burden [4]. According to data from the National Cancer Center, medical expenses caused by malignant tumors in recent years have exceeded 220 billion yuan per year, with out-of-pocket expenses accounting for more than half of total household income [5]. Although Innovative Anti-Cancer Drugs (IACDs) developed in recent years have significantly extended the survival of cancer patients, reduced pain levels, and improved their quality of life [6, 7], they are largely inaccessible to patients due to high research and development costs, patent protection, uneven regional economic development, unequal diagnostic and treatment capabilities of medical institutions, and policy promotion of pharmaceutical companies [8, 9].

Therefore, the Chinese government has implemented some specific measures. In coordination with relevant departments, the government has adopted measures such as “group purchase” to exchange price for quantity, rational drug use, medical insurance payment, and R&D innovation on the basis of reducing the tax rate [10]. To establish a long-term mechanism to control the cost burden of drugs, the Chinese government organized seven rounds of national pricing negotiations for the national reimbursement drug list (NRDL) from 2016 to the end of 2022 [11]. In July 2017, the Ministry of Human Resources and Social Security of the People’s Republic of China organized the second negotiation for drugs included in the medical insurance catalog, which was also the first national-level negotiation for innovative drugs in China. Eighteen anti-cancer medicines were included in the NRDL, thus marking the implementation phase of the national medical insurance negotiation [12]. In 2020, 162 drugs were included in the price negotiations, and more than 60% of these drugs, including 17 IACDs, were added to the list [12]. The total number of IACDs in NRDL increased from 2 in 2016 to 52 in 2020.

Multiple studies have confirmed that including IACDs in medical insurance coverage can alleviate the economic burden on patients [13, 14], reduce their out-of-pocket expenses [15], lower mortality rates [16], and expand the scope of patients’ utilization of health services [17, 18]. Studies conducted in other countries suggest that the implementation of differentiated pricing policies, stronger health technology assessments, scientific allocation of budgets, enhanced government negotiations, and centralized procurement can improve the implementation of medical insurance coverage for IACDs [19–21]. However, the lack of empirical evidence has affected the generalizability of the research results.

Until now, the effectiveness of the National Health Insurance Coverage (NHIC) policy for IACDs was evaluated based on national and institutional procurement data. However, macro evaluation based on procurement data lacks the micro perspective on individual patient characteristics. Guan et al. used national hospital procurement data to analyze the impact of pricing policies on the prices and usage of 52 IACDs [22]. Cai et al. analyzed drug procurement data from 1039 hospitals between October 2017 and December 2019 and found that after including negotiated drug prices in the national healthcare insurance program, the average availability of 17 negotiated anti-cancer drugs increased by 25.22% [18]. However, data obtained from a specific medical institution on the other hand cannot provide an overview of the actual implementation effects of local medical insurance policies. Diao et al. conducted multivariate analyses with a binary logistic regression model to analyze the impact of government health insurance coverage on drug utilization, and the determinants of medication choice among patients receiving data from a tertiary hospital in Fujian Province [23]. Xia et al. analyzed the effect of health insurance policy on the use of trastuzumab in Jiangsu Province by enrolling HER2-positive EBC patients diagnosed at 7 representative hospitals [24].

In recent years, there have been groundbreaking advances in systemic treatment for advanced hepatocellular carcinoma (HCC). Lenvatinib is a tyrosine kinase receptor inhibitor that can inhibit the kinase activity of vascular endothelial growth factor receptors [25, 26]. It was launched in China in September 2018 and was included in the NRDL as a IACD for advanced HCC in March 2021 [27]. The price of lenvatinib was reduced by 80.7% after the NHIC policy implementation [27]. The inclusion of IACDs in medical insurance coverage requires long-term evaluation of its effectiveness and continuous adjustment. To fill the gap in the policy assessment on the implementation of the NHIC policy for IACDs in typical regions in China, we evaluated the changes in the use of lenvatinib within a specific medical insurance region from the micro perspective of individual patient characteristics. The study aimed to determine the impact of the NHIC policy on the use of IACDs, and ultimately provide policy recommendations for improving the national medical insurance system and related drug policies.

## Methods

### Study design

This study determined the impact of the NHIC policy on the use of lenvatinib from the perspectives of patient characteristics. We collected continuous monthly data of adult patients with advanced HCC from September 2019 to August 2022 for 36 months. In other words, we analyzed the data for the 18 months before and after the implementation of the NHIC policy for lenvatinib. All eligible patients were divided into an exposed group and a non-exposed group according to the implementation time of the NHIC policy. The inclusion criteria for the patients were age ≥ 18 years and pathologically confirmed advanced HCC. To mitigate potential selection bias, HCC patients with incomplete data (e.g. demographic characteristics, medication information, hospitalization expenses) were excluded. All eligible patients with advanced HCC treated with lenvatinib as the first-line treatment were included in this study.

Contingency table chi-square statistics and binary logistic regression analysis were used to compare the differences in the categorical variables. Interrupted time-series (ITS) regression analysis was performed to evaluate the changes in the utilization of lenvatinib over the 36 months. Multiple linear regression was used to analyze the impact of receiving lenvatinib on total hospitalization expenses of hospitalized patients with advanced HCC.

### Type of insurance systems

**Fig. 1:**
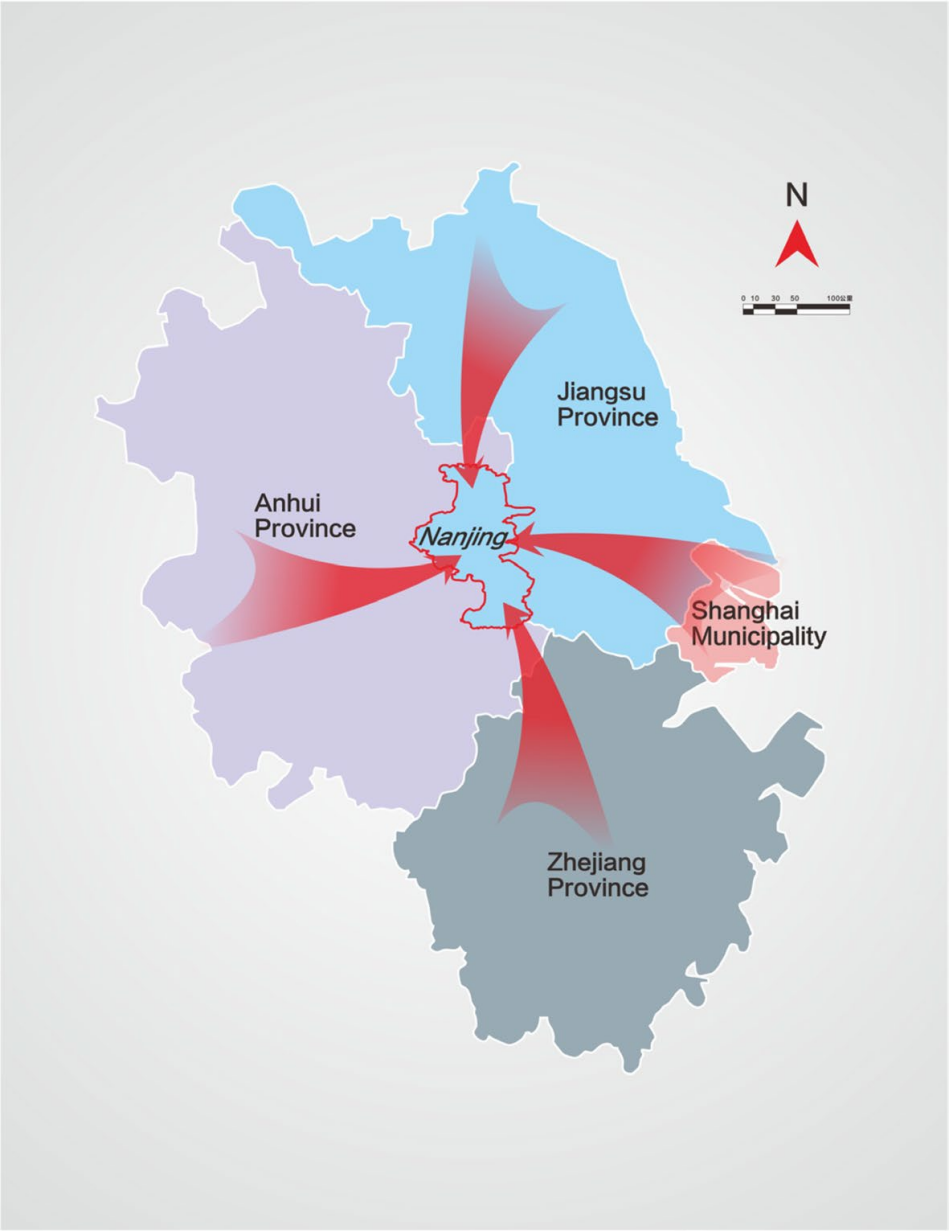
Map of 3 provinces and 1 municipality

This study was conducted in Nanjing, China. Nanjing is the capital city of Jiangsu Province and an important central city in eastern China. The Nanjing area has abundant high-quality medical resources, mainly benefiting patients from Anhui Province, Zhejiang Province, Jiangsu Province, and Shanghai City (Fig. 1). In April 2019, National Healthcare Security Administration and the Bureau of Finance jointly issued a document requesting that China realize the integration of the basic medical insurance for urban residents and the new rural cooperative medical care system into a unified urban and rural residents’ medical insurance system by the end of 2019. In January 2019, the basic medical insurance for urban residents was canceled in Nanjing, and the rural cooperative medical insurance system was abolished. Therefore, since 2019, Nanjing has mainly included two categories of social insurance systems: urban employees’ basic medical insurance (UEBMI), and urban-rural residents’ basic medical insurance (URRBMI). In March 2021, lenvatinib was included in the reimbursement drug list of Nanjing for the treatment of patients with advanced HCC according to the national price negotiation and health insurance access policy. The price was reduced from US$2498 to US$482 and the out-of-pocket ratio of UEBMI and URRBMI was 0.1.

### Data source

The data of advanced HCC patients was retrieved from the Medical and Health Big Data Center (Nanjing Health Information Platform) built by Nanjing Health Information Center. The platform integrates medical and public health data from nearly 200 medical and health institutions in the Nanjing area. The database includes general demographic characteristics, pathology and imaging reports, type of medical insurance, length of stay, hospital visit, the total hospitalization expenses, medication information, etc.

### Relevant drug

According to the guidelines of the Chinese Society of Clinical Oncology (CSCO), only sorafenib and lenvatinib as the first-line IACDs for advanced HCC have been included in the NRDL before 2022. Similar to lenvatinib, sorafenib is also a multi-targeted tyrosine kinase inhibitor [26]. In addition, sorafenib is the first IACD approved for first-line treatment of advanced HCC. It was launched in China in August 2009 and included in the NRDL in September 2017 [27]. A global multicenter REFLECT III clinical trial [26] conducted in 2018 showed that lenvatinib is not inferior to sorafenib in terms of efficacy, particularly in significantly prolonging the time to disease progression in patients. Both sorafenib and lenvatinib are limited to the reimbursement scope for patients with unresectable HCC who have not received previous systemic treatment. Therefore, sorafenib can be considered as a relevant drug that influences the use of lenvatinib. In the real world, doctors often recommend lenvatinib for patients who have poor clinical efficacy with sorafenib. Therefore, we consider sorafenib as a relevant drug to further analyze the usage of lenvatinib.

### Data management and variable definition

The following variables were collected: (i) demographic data – gender, age, occupation (peasants, administrative clerk, staff without fixed jobs, retiree, freeman), place of residence (urban or rural), division of provinces and cities (Nanjing area, other areas of Jiangsu Province except for Nanjing area, outside of Jiangsu Province); (ii) type of insurance systems - UEBMI, URRBMI, uninsured (implying payment with personal funds); (iii) length of stay, medical record data (e.g. pathology reports, imaging reports, and blood biochemistry test results), and hospitals.

### Statistical analysis

Descriptive statistics were used to characterize the proportion of patients. Contingency table chi-square statistics and binary logistic regression analysis were used to compare categorical variables and the results were reported as odds ratios (ORs) and 95% confidence intervals (95% CI). The usage of lenvatinib was defined as the dependent variable and designated as 1 when the patient received lenvatinib, and 0 otherwise. The independent variables with *P* < 0.1 in the contingency table chi-square test were included in the binary logistic regression. The “enter” method was used to fit the regression model. The utilization percentages were calculated as the proportion of all eligible patients who used lenvatinib every month during the study period. ITS regression analysis was used to evaluate the changes in the utilization of lenvatinib over the 36 months period.

$$The\;monthly\;proportion\;of\;lenvatinib\;usage\:=\:Number\;of\;patients\;who\;received\;lenvatinib\;/\;Total\;number\;of\;patients\;were\;included\;\ast\;100\%$$

**Fig. 2:**
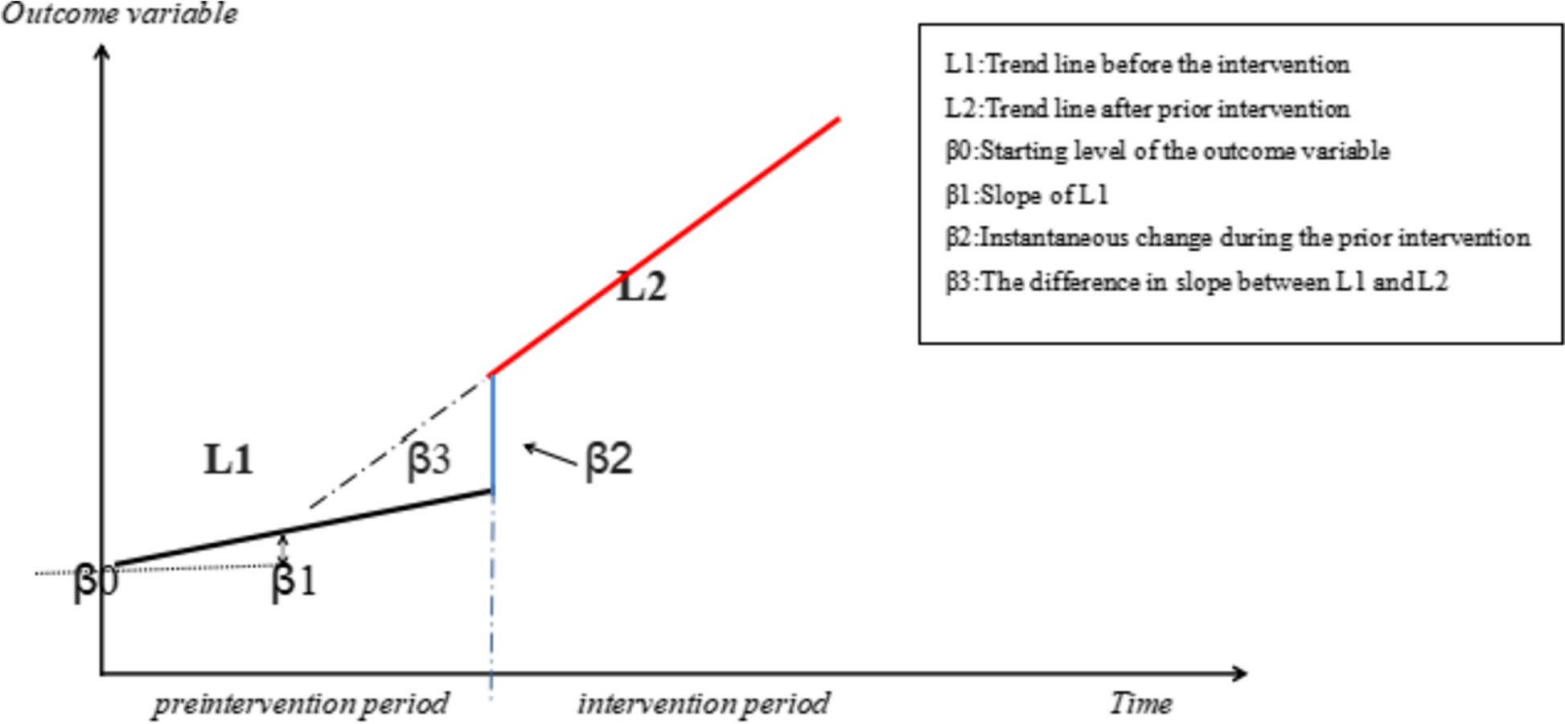
Graphic illustration of the ITS model and the trend lines for data points

The regression equation is as follows (Fig. 2):

$$Yt\mathit=\mathit\;\beta\mathit0+\mathit\;\beta\mathit1\mathit\ast time+\mathit\;\beta\mathit2\mathit\ast intervention+\mathit\;\beta\mathit3\mathit\ast post\mathit\;time+\varepsilon t$$

*Y*_*t*_ is the aggregated outcome variable measured at each equally spaced time point t, *time* is the time since the start of the study, *intervention* is a dummy (indicator) variable representing the intervention (preintervention periods 0, otherwise 1), *posttime* is the time after intervention number variable [11]. β0 represents the intercept and starting level of the outcome variable, β1 is the slope or trajectory of the outcome variable until the introduction of the prior intervention, β2 is the change in the level of the outcome that occurs in the period immediately following the introduction of the intervention (compared with a preintervention period), β3 is the difference between preintervention and the prior intervention slopes of the outcome, *Ɛt* is the residual at time t, which represents the variation of the outcome variable not explained by the model [11].

The interrupted linear regression model requires that the outcome variable has a linear trend over time before and after the intervention and that the series has no autocorrelation. The Durbin-Watson (D-W) method was used to test for the existence of 1st order autocorrelation in the time series, with values close to 2 or 4 indicating no autocorrelation. The generalized least square estimator (GLSE) was used to correct any autocorrelation. The databases and plots were constructed using Excel 2020, and STATA v.16 software was used for statistical analysis.

Multiple linear regression analysis was used to predict the changes in the total hospitalization expenses and to identify the factors associated with the use of lenvatinib. The total hospitalization expense is a quantitative variable. Therefore, a normality test was first conducted to determine whether the data conformed to a normal distribution, and then a multiple linear regression equation was established. We used variance inflation factor (VIF) tests to validate whether the multiple linear regression model had multicollinearity. VIF value less than 10 indicates the absence of multicollinearity issues and a well-constructed model, whereas VIF greater than 10 indicates that the model is poorly constructed. When the Beta value is greater than 0, it has a positive impact, and the larger the value, the greater the impact. When the Beta value is less than 0, it has a negative impact, and the smaller the value, the greater the impact. SPSS 26.0 (IBM Corp., Armonk, NY) and STATA v.16 (Stata Corp, LLC, College Station, Texas) were used for the above analyses. The statistical significance level was set at *P* < 0.05.

$$Total\;hospitalization\;expenses\:=\:The\;sum\;of\;all\,costs\;incurred\;during\;hospitalization\;(Note:\;the\;sum\;of\;all\;costs\;consisted\;of\;out-of-pocket\,costs\;and\;insurance-covered\;cost).$$

## Results

### Patient characteristics

From September 2019 to August 2022, 12,659 patients with advanced HCC were included in this study, including 9803 (77.44%) males and 2856 (22.56%) females. The number of hospitalized patients with HCC was respectively 5884 and 6775 in the 36 months before and after the NHIC policy (Table 1). The usage rate of lenvatinib increased from 6.19% before the NHIC policy to 15.28% after the NHIC policy. The mean age of patients was 60.27 ± 11.00 years, and the highest proportion of patients was between 50 and 65 years old (47.77%). In addition, 9351 patients used medical insurance, of which URRBMI was the most common (76.06%). Peasants comprised the largest proportion in terms of occupation (33.16%). Urban residents accounted for 65.39% of the patients, and most were from the Nanjing area (56.32%). Furthermore, 97.96% of the patients were hospitalized in the tertiary or higher hospitals, which is related to the high diagnostic and treatment capacity of medical centers equipped for treating HCC.

**Table 1:** The baseline characteristics of HCC patients

Variable		Number	%
Gender	Male	9803	77.44
	Female	2856	22.56
Age	≥ 18, < 35	210	1.66
	≥ 35, < 50	1735	13.71
	≥ 50, < 65	6047	47.77
	≥ 65, < 80	4201	33.19
	≥ 80	466	3.68
Implementation of the NHIC policy	Before	5884	46.48
	After	6775	53.52
Medication status	Used	1399	11.05
	Unused	11,260	88.95

### Factors associated with the use of lenvatinib

The usage rate of lenvatinib increased from 6.19% to 15.28% over the 36 months period from September 2019 to August 2022, and the difference was statistically significant (*P* < 0.001; Table 2). We performed binary logistic regression analysis to identify the factors associated with the use of lenvatinib. Based on the results of the contingency table chi-square test, gender, age, type of insurance system, occupation, place of residence, division of provinces and cities, and hospital level were included in the regression model. By controlling the other factors, the probability of patients with advanced HCC receiving lenvatinib increased by 2.72-fold after the policy implementation (OR = 2.720, 95% CI: 2.396–3.088, *P* < 0.001). In addition, the use of lenvatinib was significantly associated with all six variables (*P* < 0.05). However, no significant difference was observed in the probability of usage across different provincial/municipal regions (*P* = 0.644 for other areas of Jiangsu Province except Nanjing area and *P* = 0.978 for outside of Jiangsu Province; Table 3). Before March 2021, the utilization rate of sorafenib was 8.70%, and decreased to 6.91% (-1.79%) after the inclusion of lenvatinib. In fact, there was a significant association between the use of sorafenib and lenvatinib (*P* < 0.001). Furthermore, patients who had used sorafenib were more likely to choose lenvatinib compared to those who were not treated with sorafenib (OR = 2.021, 95% CI:1.705–2.397, *P* < 0.001).

**Table 2:** Contingency table chi-square test of the factors associated with the use of lenvatinib

Patient characteristics		Number (%)		*χ* ^*2*^	*P*
		Unused	Used		
**Gender**	Male	8656 (88.30)	1147 (11.70)	18.622	0.001
	Female	2604 (91.18)	252 (8.82)		
**Age**	≥ 18, < 35	176 (83.81)	34 (16.19)	15.598	0.004
	≥ 35, < 50	1519 (87.55)	216 (12.45)		
	≥ 50, < 65	5364 (88.71)	683 (11.29)		
	≥ 65, < 80	3776 (89.88)	425 (10.12)		
	≥ 80	425 (91.20)	41 (8.80)		
**Type of insurance system**	UEBMI	1916 (85.57)	323 (14.43)	64.378	<0.001
	URRBMI	6463 (90.87)	649 (9.13)		
	Uninsured	2881 (87.09)	427 (12.91)		
**Occupation**	Peasants	3759 (89.54)	439 (10.46)	36.552	<0.001
	Administrative clerk	871 (83.83)	168 (16.17)		
	Staff without fixed jobs	2547 (89.62)	295 (10.38)		
	Retiree	1064 (87.28)	155 (12.72)		
	Freeman	3019 (89.82)	342 (10.18)		
**Location of residence**	Urban	7332 (88.57)	946 (11.43)	3.448	0.063
	Rural	3928 (89.66)	453 (10.34)		
**Division of provinces and cities**	Nanjing area	6389 (89.62)	740 (10.38)	10.756	0.005
	Other areas of Jiangsu Province except Nanjing area	3203 (87.54)	456 (12.46)		
	Outside of Jiangsu Province	1668 (89.15)	203 (10.85)		
**Hospital level**	The tertiary and above hospital	11,009 (88.78)	1392 (11.22)	18.628	<0.001
	Below the tertiary level	251 (97.29)	7 (2.71)		
**Implementation of the NHIC policy**	Before	5520 (93.81)	364 (6.19)	264.728	<0.001
	After	5740 (84.72)	1035 (15.28)

**Table 3:** The binary logistic regression analysis results of the associated factors of patients who received lenvatinib

Patient characteristics		*P*	*OR*	*95% CI*	
**Gender**	Female	<0.001	0.743	0.642	0.860
	Male		Ref		
**Age**	≥ 18, < 35	<0.001	2.522	1.504	4.229
	≥ 35, < 50	0.019	1.582	1.079	2.318
	≥ 50, < 65	0.066	1.388	0.978	1.971
	≥ 65, < 80	0.601	1.095	0.779	1.540
	≥ 80		Ref		
**Hospital level**	Below the tertiary level	<0.001	0.252	0.118	0.538
	The tertiary and above hospital		Ref		
**Type of insurance system**	UEBMI	0.187	2.119	0.695	6.464
	URRBMI	<0.001	0.755	0.651	0.875
	Uninsured		Ref		
**Occupation**	Administrative clerk	0.195	0.477	0.155	1.463
	Staff without fixed jobs	0.010	0.661	0.482	0.907
	Retiree	0.177	0.455	0.145	1.427
	Freeman	0.301	0.845	0.613	1.163
	Peasants		Ref		
**Location of residence**	Rural	0.023	0.722	0.544	0.957
	Urban		Ref		
**Division of provinces and cities**	Other areas of Jiangsu Province except Nanjing area	0.644	1.039	0.884	1.222
	Outside of Jiangsu Province	0.978	0.997	0.816	1.218
	Nanjing area		Ref		
**Implementation of the NHIC policy**	After	<0.001	2.720	2.396	3.088
	Before		Ref		

We also analyzed the factors associated with lenvatinib use before and after policy implementation to evaluate its potential impact. Before the implementation of the NHIC policy, there was a significant correlation between the use of lenvatinib and five variables, including age, type of insurance systems, occupation, place of residence, and division of provinces and cities (*P* < 0.05). After policy implementation, the probability of lenvatinib use was significantly higher among male patients and in hospitals at or above the tertiary level, compared to female patients and in hospitals below the tertiary level respectively (*P* < 0.05). In addition, younger patients are more likely to receive lenvatinib. The probability of lenvatinib use among patients in Nanjing City is opposite to that before the policy was implemented, and is lower than that of inpatients in other regions of Jiangsu Province (OR = 1.294, 95% CI:1.075–1.557, *P* = 0.006; Table 4).

**Table 4:** The associated factors of patients who received lenvatinib before and after the NHIC policy

Patient characteristics		Before the policy				After the policy			
		*P*	*OR*	*95% CI*		*P*	*OR*	*95% CI*	
**Gender**	Female	0.067	0.765	0.575	1.019	0.001	0.745	0.627	0.884
	Male		Ref				Ref		
**Age**	≥ 18, < 35	0.021	2.470	1.149	5.310	0.001	3.440	1.673	7.073
	≥ 35, < 50	0.621	0.859	0.470	1.569	<0.001	2.887	1.689	4.935
	≥ 50, < 65	0.659	0.892	0.536	1.484	0.001	2.288	1.380	3.794
	≥ 65, < 80	0.101	0.665	0.408	1.083	0.015	1.849	1.125	3.038
	≥ 80		Ref				Ref		
**Hospital level**	Below the tertiary level	0.074	0.467	0.203	1.077	0.005	0.061	0.008	0.438
	The tertiary and above hospital		Ref				Ref		
**Type of insurance system**	UEBMI	0.912	1.078	0.285	4.081	0.224	2.985	0.512	17.385
	URRBMI	<0.001	0.325	0.246	0.430	0.387	1.081	0.907	1.287
	Uninsured		Ref				Ref		
**Occupation**	Administrative clerk	0.102	0.321	0.082	1.255	0.405	0.472	0.081	2.765
	Staff without fixed jobs	<0.001	0.258	0.126	0.529	0.542	0.896	0.629	1.276
	Retiree	0.079	0.279	0.067	1.161	0.413	0.473	0.079	2.832
	Freeman	0.052	0.495	0.243	1.007	0.893	0.975	0.679	1.401
	Peasants		Ref				Ref		
**Location of residence**	Rural	0.002	0.364	0.190	0.699	0.424	0.880	0.642	1.205
	Urban		Ref				Ref		
**Division of provinces and cities**	Other areas of Jiangsu Province except Nanjing area	0.001	0.560	0.392	0.801	0.006	1.294	1.075	1.557
	Outside of Jiangsu Province	0.342	0.837	0.580	1.208	0.601	1.066	0.838	1.358
	Nanjing area		Ref				Ref		

Before the implementation of the NHIC policy, only patients younger than 35 years had a higher probability of receiving lenvatinib (*P* = 0.021). After the policy was implemented, the probability of receiving lenvatinib increased significantly for patients in all age groups compared to those aged 80 or above (*P* < 0.05). There was no significant difference in the probability of lenvatinib usage among patients hospitalized in hospitals of different levels before the policy was implemented. However, patients hospitalized in tertiary or higher-level hospitals were more likely to receive lenvatinib compared to those admitted to hospitals below the tertiary level after policy implementation (*P =* 0.005). Furthermore, the probability of receiving lenvatinib among rural patients was only 0.364 times that of urban patients before the NHIC policy (OR = 0.364, 95% CI:0.190–0.699, *P* = 0.002; Table 4), whereas the implementation of the policy has lessened the gap among patients from different residential areas.

### ITS analysis of changes in the trend of utilization

**Fig. 3:**
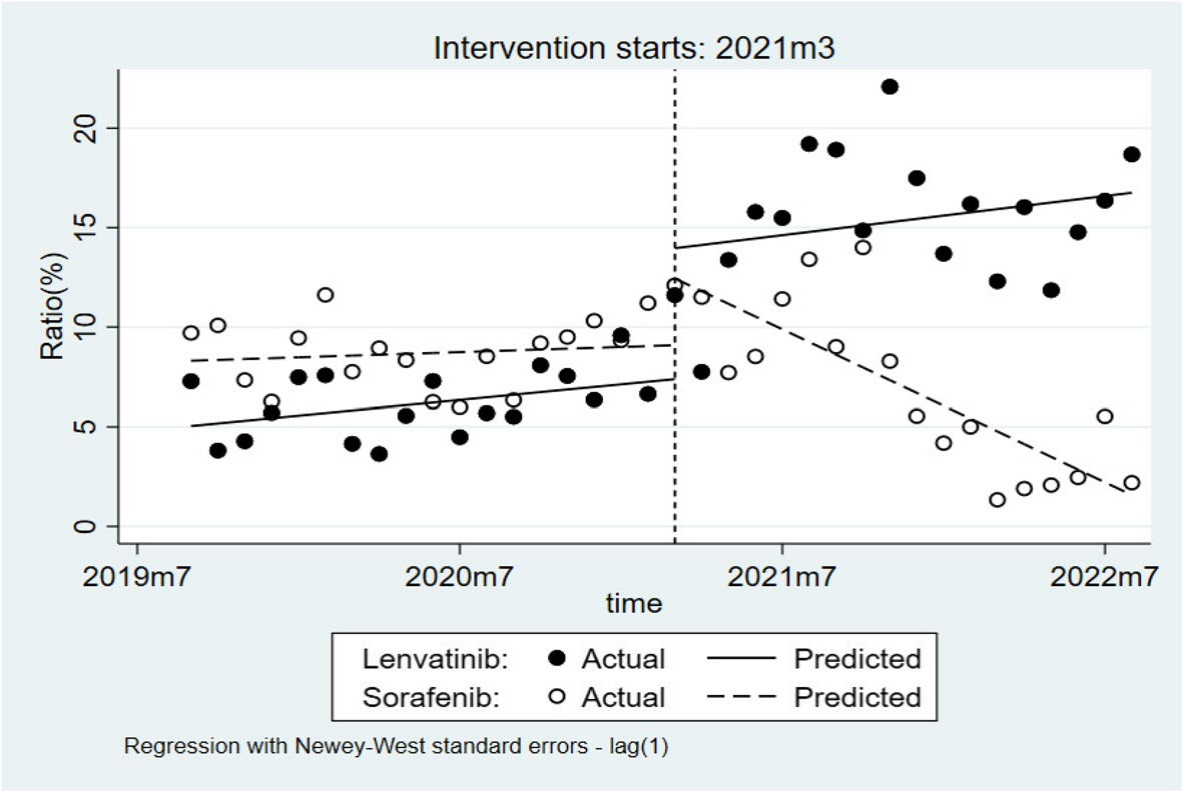
Results of the regression analysis of the monthly proportion of lenvatinib and sorafenib usage

To further evaluate the trend in lenvatinib and sorafenib utilization before and after the policy change, and to assess the immediate and long-term effects of the policy, we selected 18 monthly time points before and after its implementation (a total of 36 months), and used segmented regression models in ITS. After the policy implementation, the utilization rate of lenvatinib showed an upward trend, and the time-dependent effect was significant (*P* = 0.003). In contrast, the utilization rate of sorafenib showed a downward trend, and the change was statistically significant (*P* < 0.001; Table 5). As shown in Fig. 3, the utilization rate of lenvatinib increased from less than 10% to around 20%, while that of sorafenib decreased from around 10% to less than 5%.

**Table 5:** Estimates the impact of the NHIC policy on the monthly proportion of usage

Variable	Lenvatinib				Sorafenib			
	*β*	*t*	*P*	*95%CI*	*β*	*t*	*P*	*95%CI*
β0	5.173	4.760	<0.001	2.959 ~ 7.386	8.333	8.000	<0.001	6.211 ~ 10.454
β1	0.137	1.300	0.203	-0.078 ~ 0.352	0.070	0.740	0.465	-0.123 ~ 0.264
β2	6.443	3.210	0.003	2.354 ~ 10.532	2.857	1.970	0.057	-0.096 ~ 5.810
β3	0.039	0.190	0.848	-0.371 ~ 0.448	-0.702	-4.450	<0.001	-1.024~-0.381

### Impact of lenvatinib use and other factors on the total hospitalization expenses among patients with advanced HCC after the NHIC policy

The results showed that there was multicollinearity between 3 categorical indicators of 2 variables (occupation, type of insurance systems), with VIF values of 52.76, 32.86, and 27.84. To eliminate the impact of multicollinearity, the occupation variable was deleted. Thereafter, a significant impact relationship (*P* < 0.05) was observed between 5 variables (gender, division of provinces and cities, hospital level, the NHIC policy, and lenvatinib use), and the total hospitalization expenses of patients. The degree of influence of these variables on the total hospitalization expense was determined based on Beta values, and the factors in order of influence were hospital level (Beta = 0.105), lenvatinib use (Beta = 0.062), division of provinces and cities (Beta=-0.055), NHIC policy (Beta=-0.040), and gender (Beta=-0.025). After the implementation of the NHIC policy, the total hospitalization expenses for patients increased (US$4780.93 ± US$5916.34 vs. US$4946.14 ± US$4869.48, Beta=-0.040, *P* < 0.001). Compared to patients who received lenvatinib, the total hospitalization expenses were higher for those who did not use the drug (US$5022.07 ± US$5488.70 vs. US$3701.63 ± US$4330.70, Beta = 0.062, *P* < 0.001; Table 6).

**Table 6:** Multiple linear regression analysis results of the associated factors of the total hospitalization expenses

Patient characteristics		Standardized coefficients *(Beta)*	*t*	*P*	*VIF*
Gender	Male	-0.025	-2.690	0.007	1.02
	Female	Ref			
Age	≥ 18, < 35	-0.005	-0.480	0.630	1.46
	≥ 35, < 50	-0.007	-0.370	0.714	4.27
	≥ 50, < 65	-0.036	-1.440	0.151	7.64
	≥ 65, < 80	0.017	0.700	0.482	6.93
	≥ 80	Ref			
Hospital level	The tertiary and above hospital	0.105	11.420	<0.001	1.01
	Below the tertiary level	Ref			
Type of insurance system	UEBMI	-0.016	-1.420	0.156	1.52
	URRBMI	-0.009	-0.720	0.471	1.68
	Uninsured	Ref			
Location of residence	Urban	0.020	1.690	0.091	1.69
	Rural	Ref			
Division of provinces and cities	Nanjing area	-0.055	-3.480	0.001	3.01
	Other areas of Jiangsu Province except Nanjing area	0.014	1.050	0.292	2.09
	Outside of Jiangsu Province	Ref			
Lenvatinib	Unused	0.062	6.700	<0.001	1.03
	Used	Ref			
Implementation of the NHIC policy	Before	-0.040	-4.280	<0.001	1.05
	After	Ref			

## Discussion

We conducted a retrospective analysis to address the effects of implementing the NHIC policy for IACDs in typical regions of China. The impact was analyzed from the perspectives of macro-drug accessibility and micro-patient individual characteristics.

### The NHIC policy has increased the likelihood of advanced HCC patients receiving lenvatinib

The percentage of HCC patients receiving lenvatinib increased from 6.19% before the NHIC policy to 15.28% after policy implementation. The probability of patients with advanced HCC receiving lenvatinib increased by 2.72-fold after the policy implementation. The NHIC policy has significantly increased the utilization of IACDs, and the results were consistent with the conclusions of the papers published by our research group in 2022 [18]. Based on a previous study conducted on patients with early-stage breast cancer from 7 representative hospitals in Jiangsu Province, a study concluded that the utilization rate of IACDs had grown markedly after the implementation of the NHIC policy [24]. Before lenvatinib was included in the NDRL, sorafenib was the only IACD included in the NDRL for the treatment of advanced HCC [28, 29]. After the inclusion of lenvatinib in March 2021, the utilization rate of sorafenib decreased by 1.79%. Likewise, the ITS analysis showed an upward trend in the utilization rate of lenvatinib, and a downward trend for sorafenib. According to prior research, lenvatinib has higher cost-effectiveness compared with sorafenib in the treatment of patients with HCC at the current economic level in China [30, 31]. This suggests that in the first-line treatment of advanced HCC, there is a clear substitutable relationship between lenvatinib and sorafenib. In addition, we found that patients who had previously used sorafenib were more likely to choose lenvatinib compared to those who were not treated with sorafenib. This may be related to the economic status of the patients as well as the medication habits of clinicians.

### Factors affecting the implementation effect of the NHIC policy

The probability of lenvatinib use was lower in female patients compared to male patients, which can be easily attributed to the higher incidence of HCC among males. The male-to-female ratio of HCC incidence in China ranges from 2:1 to 5:1 [1]. Furthermore, compared with patients aged 80 years and above, the proportion of patients using lenvatinib in other ages has significantly increased, which may be related to factors such as the underlying diseases, the complexity of disease course, and the low physical tolerance for older patients [30, 32]. Using the policy implementation time as the node, we conducted a stratified analysis. Overall, our results indicate that the NHIC policy has increased accessibility and the utilization of lenvatinib among all age groups. Diao et al. obtained similar conclusions from the data on breast cancer patients from one of the tertiary public hospital in the Fujian Province of China [23], but the conclusions they received were not as evident as the conclusion of our big data study.

Patients living in rural areas had a lower probability of receiving lenvatinib compared to urban patients. But, we conclude that the gap between urban and rural patients is narrowing. The improved drug accessibility for rural patients as a result of the NHIC policy indirectly suggests increased affordability in this group. Due to the difference in per capita disposable income and ratios of individual payment needed between urban and rural residents, the latter often have a higher health economic burden [33, 34]. As we know, multiple clinical examinations, chemotherapy over a long period, and standard tests incur high total costs. Therefore, a multi-level medical security system should be in place to top up the basic cover offered by the NHIC policy. Huang et al. researched non-Hodgkin lymphoma patients covered by different medical insurance types. They did not find a significant difference in the selection of IACD between insured and uninsured patients [35]. Interestingly, compared to uninsured patients in our study, the probability of lenvatinib use was lower in patients covered by URRBMI, whereas no significant difference was observed with patients covered by UEBMI. To figure out the reasons that the probability of patients with advanced HCC receiving lenvatinib covered by URRBMI was lower than that of uninsured patients, we analyzed the geographic distribution of self-paying patients. We found that 74% of self-paying patients come from areas outside of Nanjing. The cross-regional insurance reimbursement is based on the insurance list of the medical treatment location but follows the reimbursement policy of the insured area. The differences in the insured area can lead to differences in the compensation level of medical insurance [36]. Therefore, some patients may choose fully self-funded payment for their treatment at the treatment center and then be reimbursed in the insured area. In addition, since January 2019, the “new rural cooperative medical insurance” and “urban resident medical insurance” have been merged into the URRBMI [37]. Furthermore, most of the participants in the URRBMI program are peasants and unemployed urban residents with lower incomes, which explains the low drug utilization rate.

### The impact of the NHIC policy on the total hospitalization expenses

The total hospitalization expenses refer to all expenses incurred by patients during hospitalization, including drug costs, laboratory fees, treatment fees, material costs, etc. It may differ among patients treated for the same disease depending on many factors, such as length of hospital stay, complications, age, admission status, personal economic level, etc [38]. Li et al. [39] found that the shares of out-of-pocket expenditure for lung cancer treatment of the patients who were covered by resident program enrollees and formal employee program enrollees not entitled to government-funded supplementary insurance were higher than that of the patients who were covered by employee program enrollees with government-funded supplementary insurance. Shi et al. [38] obtained similar conclusions and analyzed the influencing factors of 5 types of cancer patients’ hospitalization expenses, and concluded that the total hospitalization expenses of patients covered by UEBMI were higher than those covered by URRBMI. But, we did not find a significant difference in the total hospitalization expenses of patients covered by different health insurance programs. Inspired by the above papers, a study of out-of-pocket expenses for patients with different types of health insurance will be the next plan for our group.

We conducted that the total hospitalization expenses of patients with advanced HCC increased after lenvatinib was included in NRDL, which can be directly related to the increased use of the drug. In addition, patients who do not receive lenvatinib have higher total hospitalization expenses. After conducting a more in-depth analysis, there could be two reasons. Firstly, the results of our study indicate that the majority of patients with advanced HCC who do not receive lenvatinib are elderly patients. Therefore, elderly patients with poor physical conditions may lead to higher total hospitalization expenses for patients due to the treatment of multiple complications and underlying diseases [40]. Besides, in the scope of patients who do not receive lenvatinib, some patients with severe conditions may therefore enter combined therapy. This leads to high total hospitalization expenses even without the use of lenvatinib. Therefore, the higher total hospitalization expenses for patients with advanced HCC who do not receive lenvatinib may be related to the large proportion of elderly patients and the complex disease status of patients with advanced HCC.

To summarize, the NHIC policy has significantly increased the utilization of lenvatinib, and has benefited a large number of patients with advanced HCC. In addition, being older, living in rural areas, lack of fixed income, the URRBMI coverage, and receiving treatment in hospitals below the tertiary level were the factors affecting the use of lenvatinib among patients with advanced HCC, which can be related to the patient’s health status, personal economic level, urban-rural income gap, inconsistent medical insurance coverage, and inadequacy of diagnosis and treatment conditions for cancer in primary hospitals. All the above risk factors that affect the utilization of lenvatinib prompt the importance of establishing multi-level medical insurance coverage among rural populations, low-income urban populations, and the elderly. In addition, some studies also indicated that patent protection and the policy promotion of pharmaceutical companies were the factors affecting the usage of IACDs [7, 41].

In March 2020, the Central Committee of the Communist Party of China and the State Council issued the “Opinions on Deepening the Reform of the Medical Security System”, which proposed the goal of building a multi-level medical security system. In 2018, Zhuhai City in Guangdong Province took the lead in launching the additional supplementary medical insurance program “Boundless Love”, completing the construction of a multi-level medical security system consisting of basic medical insurance, supplementary medical insurance, additional supplementary medical insurance, charity aid, and medical assistance. As of the end of 2021, the total number of insured persons under the “Boundless Love” insurance program was 833,100, with an insurance rate of 47.5% for individuals aged 60 and above. The program covered 37 self-funded anti-cancer drugs, and a personal contribution ratio of only 9.83% [42]. Overall, in response to the economic disparities between patients and between urban and rural areas, we suggest that more regions follow the example of Zhuhai City and establish multi-level medical insurance systems for economically disadvantaged patients. Finally, the total hospitalization expenses are influenced by multiple factors.

To the best of our knowledge, this is the first study to use medical big data to analyze the impact of medical insurance policies on the use of lenvatinib from the perspectives of patient characteristics, medication utilization rates, and total hospitalization expenses. It verifies the scientific and extrapolative nature of medical insurance coverage policies for IACDs in typical regions in the real world. However, this study has certain limitations that ought to be considered. First, we only analyzed information on drug usage among hospitalized patients and didn’t include outpatient data, which may have led to an underestimation of drug utilization. In addition, due to the limited information collected at baseline, we cannot characterize other factors such as stage, previous treatment, comorbidity, hepatitis B or C infection, dosage pattern, duration of treatment, and mode of therapy. Second, the study lacked qualitative interviews with patients and physicians, which affects the extensionality of the research findings. Third, we analyzed the medication choice of the patients without considering the clinicians’ preferences. However, cancer diagnosis and treatment do not always follow the national guidelines in China. Future studies should consider the potential impact of physician preference on the utilization of IACDs.

## Conclusions

Our findings indicated that the NHIC policy has significantly increased, while other factors included older, residency in rural areas, lack of fixed income decreased the utilization of lenvatinib, it is crucial to improve the multi-level medical insurance coverage for economically disadvantaged patients. The multi-level medical security systems include, but is not limited to, basic medical insurance, medical assistance, serious illness insurance, and commercial insurance jointly established by the government, enterprises, the market, and individuals [43, 44].

## Data Availability

Data are available on request. The data sets used and analysed during the current study are available from the corresponding author on reasonable request.

## Abbreviations

NRDL: Negotiations for the national reimbursement drug list
HCC: Hepatocellular carcinoma
ITS: Interrupted time-series
NHIC: National Health Insurance Coverage
IACDs: Innovative Anti-Cancer Drugs
CSCO: Chinese Society of Clinical Oncology
UEBMI: Urban employees’basic medical insurance
URRBMI: Urban-rural residents’basic medical insurance
ORs: Odds ratios
D-W: Durbin-Watson
GLSE: Generalized least square estimator
VIF: Variance inflation factor
